# COVID-19 Vaccination Coverage and Intent Among Adults Aged 18–39 Years — United States, March–May 2021

**DOI:** 10.15585/mmwr.mm7025e2

**Published:** 2021-06-25

**Authors:** Brittney N. Baack, Neetu Abad, David Yankey, Katherine E. Kahn, Hilda Razzaghi, Kathryn Brookmeyer, Jessica Kolis, Elisabeth Wilhelm, Kimberly H. Nguyen, James A. Singleton

**Affiliations:** ^1^CDC COVID-19 Response Team; ^2^Leidos, Inc., Atlanta, Georgia.

Since April 19, 2021, all persons aged ≥16 years in the United States have been eligible to receive a COVID-19 vaccine. As of May 30, 2021, approximately one half of U.S. adults were fully vaccinated, with the lowest coverage and lowest reported intent to get vaccinated among young adults aged 18–39 years (*1**–**4*). To examine attitudes toward COVID-19 vaccination and vaccination intent among adults in this age group, CDC conducted nationally representative household panel surveys during March–May 2021. Among respondents aged 18–39 years, 34.0% reported having received a COVID-19 vaccine. A total of 51.8% were already vaccinated or definitely planned to get vaccinated, 23.2% reported that they probably were going to get vaccinated or were unsure about getting vaccinated, and 24.9% reported that they probably or definitely would not get vaccinated. Adults aged 18–24 years were least likely to report having received a COVID-19 vaccine and were most likely to report being unsure about getting vaccinated or that they were probably going to get vaccinated. Adults aged 18–39 years with lower incomes, with lower educational attainment, without health insurance, who were non-Hispanic Black, and who lived outside of metropolitan areas had the lowest reported vaccination coverage and intent to get vaccinated. Concerns about vaccine safety and effectiveness were the primary reported reasons for not getting vaccinated. Vaccination intent and acceptance among adults aged 18–39 years might be increased by improving confidence in vaccine safety and efficacy while emphasizing that vaccines are critical to prevent the spread of COVID-19 to friends and family and for resuming social activities (*5*).

During March–May 2021, CDC sponsored questions in two nationally representative, probability-based panel surveys (Ipsos Knowledge Panel and NORC AmeriSpeak)* that were administered to U.S. adults aged ≥18 years to assess COVID-19 vaccination status, intent, attitudes, and perceptions (*6**–**8*). Eight surveys were administered to 8,410 panelists (approximately 1,000 per panel) during March 5–May 2, 2021, with panel completion^†^ ranging from 20.3% to 60.1%. Because of similar sampling methods and characteristics of respondents, results were pooled across surveys.

For each survey, respondents were asked questions about receipt of COVID-19 vaccine and intent to get vaccinated if not already vaccinated, as well as questions about their perceptions of the COVID-19 vaccine. Respondents were asked, “Have you received a COVID-19 vaccine?” and those who answered “no” were asked, “Once a vaccine to prevent COVID-19 is available to you, would you: definitely get a vaccine, probably get a vaccine, be unsure about getting a vaccine, probably not get a vaccine, or definitely not get a vaccine?” Respondents were grouped by vaccination and intent status as follows^§^: 1) persons who had already received a COVID-19 vaccine or who were definitely intending to get vaccinated; 2) persons who were probably getting vaccinated or who were unsure about getting vaccinated; and 3) persons who probably or definitely did not intend to get vaccinated.

Analyses were conducted among the subset of adults aged 18–39 years (N = 2,726) to estimate vaccination coverage and intent by sociodemographic characteristics^¶^ and to assess COVID-19 vaccine perceptions among intent groups. All survey data were weighted to U.S. Census geodemographic benchmarks to ensure representativeness and analyzed using SAS-callable SUDAAN (version 11.0.1; RTI International). T-tests were used to determine differences by age and sociodemographic characteristics. This activity was reviewed by CDC and was conducted consistent with applicable federal law and CDC policy.**

Among 2,726 adults aged 18–39 years, 51.8% reported that they had been vaccinated or were definitely intending to get vaccinated, including 34.0% who had already received a COVID-19 vaccine; 23.2% were probably going to get vaccinated or were unsure about getting vaccinated; and 24.9% reported that they probably or definitely would not get vaccinated (Table 1). Compared with adults aged 35–39 years, a smaller proportion of adults aged 18–24 years reported having been vaccinated (28.4% versus 35.5%), and a larger proportion was unsure about getting vaccinated or was probably going to get vaccinated (28.3% versus 19.2%).

**TABLE 1:** COVID-19 vaccination and intent status among adults aged 18–39 years, by sociodemographic characteristics — United States, March–May 2021

Characteristic	Total no.*	% (95% CI)	Weighted % (95% CI^†^)			
			Vaccination status	Vaccination and intent status		
			Vaccinated (N = 1,022)	Vaccinated or definitely planning to get vaccinated (N = 1,521)	Unsure or probably will get vaccinated (N = 562)	Probably or definitely will not get vaccinated (N = 643)
**Total**	**2,726**	**100 (99.9–100.0)**	**34.0 (31.9–36.2)**	**51.8 (49.3–54.4)**	**23.2 (21.1–25.4)**	**24.9 (22.9–27.1)**
**Age group, yrs**						
18–24	**532**	**29.1 (26.8–31.4)**	28.4 (23.9–33.3)^§^	49.9 (44.7–55.1)	28.3 (23.5–33.4)^§^	21.8 (17.9–26.2)
25–29	**675**	**25.2 (23.2–27.3)**	36.1 (31.7–40.7)	50.6 (45.8–55.5)	24.6 (20.6–28.8)	24.8 (20.6–29.4)
30–34	**834**	**22.9 (21.1–24.7)**	37.4 (33.5–41.5)	54.6 (50.3–58.9)	19.4 (16.1–22.9)	26.0 (22.4–29.9)
35–39 (Ref)	**685**	**22.9 (21.0–24.7)**	35.5 (31.5–39.6)	52.8 (48.3–57.4)	19.2 (15.7–23.2)	27.9 (24.0–32.2)
**Sex**						
Female (Ref)	**1,395**	**51.2 (48.7–53.7)**	34.3 (31.2–37.4)	50.7 (47.1–54.3)	21.9 (19.2–24.9)	27.4 (24.2–30.8)
Male	**1,331**	**48.8 (46.3–51.3)**	33.8 (30.7–37.0)	53.1 (49.6–56.4)	24.6 (21.3–28.1)	22.3 (19.6–25.3)^§^
**Race/Ethnicity**						
White, non-Hispanic (Ref)	**1,684**	**54.9 (52.3–57.4)**	35.0 (32.4–37.8)	51.8 (48.8–54.8)	21.4 (18.8–24.2)	26.8 (24.2–29.5)
Black, non-Hispanic	**270**	**12.3 (10.6–14.1)**	25.4 (19.6–32.0)^§^	40.1 (33.2–47.2)^§^	27.6 (21.0–35.1)	32.3 (25.7–39.5)
Hispanic	**467**	**21.5 (19.5–23.7)**	33.7 (28.4–39.2)	52.2 (46.4–58.0)	25.8 (20.7–31.3)	22.0 (17.0–27.6)
All other races, non-Hispanic^¶^	**305**	**11.4 (9.8–13.1)**	39.0 (31.9–46.5)	63.9 (56.9–70.4)^§^	22.5 (16.9–28.9)	13.6 (8.9–19.6)^§^
**Education**						
Less than high school	**200**	**13.1 (11.2–15.2)**	16.2 (11.1–22.5)^§^	32.4 (25.0–40.5)^§^	31.8 (24.4–39.8)^§^	35.8 (27.3–45.1)^§^
High school diploma or equivalent	**533**	**28.0 (25.6–30.4)**	23.6 (19.3–28.3)^§^	40.7 (35.6–45.9)^§^	28.5 (24.0–33.3)^§^	30.8 (26.4–35.6)^§^
Some college	**932**	**28.9 (26.9–30.9)**	33.6 (30.0–37.4)^§^	49.9 (46.0–53.8)^§^	24.6 (21.2–28.4)^§^	25.5 (22.2–29.0)^§^
Bachelor’s degree or higher (Ref)	**1,061**	**30.0 (28.0–32.1)**	51.8 (48.5–55.2)	72.6 (69.4–75.7)	13.3 (11.1–15.7)	14.1 (11.7–16.8)
**Household income, $**						
<24,999	**420**	**19.1 (16.9–21.4)**	21.0 (16.2–26.3)^§^	36.2 (30.0–42.7)^§^	27.0 (21.8–32.7)^§^	36.8 (30.9–42.9)^§^
25,000–49,999	**604**	**22.2 (20.2–24.2)**	28.0 (24.0–32.3)^§^	43.8 (39.1–48.7)^§^	26.3 (21.4–31.7)^§^	29.9 (25.4–34.6)^§^
50,000–74,999	**537**	**18.4 (16.7–20.1)**	35.3 (30.4–40.5)^§^	50.5 (45.3–55.7)^§^	24.7 (20.3–29.6)	24.7 (20.3–29.6)^§^
≥75,000 (Ref)	**1,165**	**40.3 (38.0–42.7)**	42.9 (39.5–46.4)	64.2 (60.9–67.5)	19.1 (16.3–22.1)	16.7 (14.5–19.1)
**Health insurance coverage**						
Insured (Ref)	**2,272**	**84.8 (82.7–86.7)**	36.2 (33.9–38.6)	55.4 (52.7–58.1)	21.9 (19.7–24.3)	22.6 (20.5–24.9)
Not insured	**358**	**15.2 (13.3–17.3)**	24.5 (19.7–29.8)^§^	35.8^§^ (29.6–42.3)	28.3 (22.5–34.6)	36.0 (29.3–43.1)^§^
**Metropolitan residence**						
Metropolitan (Ref)	**2,338**	**84.2 (82.2–85.9)**	35.4 (33.1–37.7)	55.0 (52.3–57.7)	22.9 (20.8–25.2)	22.1 (19.9–24.4)
Nonmetropolitan	**388**	**15.8 (14.1–17.8)**	26.9^§^ (21.9–32.4)	35.0 (29.3–41.1)^§^	24.9 (18.9–31.7)	40.1 (34.0–46.4)^§^

**Abbreviations:** CI = confidence interval; Ref = referent group.

* No. = unweighted sample size/denominator.

^†^ Korn-Graubard 95% CI.

^§^ Statistically significant difference compared with referent group.

^¶^ Includes non-Hispanic Asian, American Indian or Alaska Native, Native Hawaiian or Other Pacific Islander, and multiple races.

COVID-19 vaccination and intent differed by demographic characteristics (Table 1). Education and income were both associated with likelihood of vaccination and all levels of intent. Those with a bachelor’s degree or higher were most likely to report being vaccinated or definitely intending to get vaccinated (72.6%), including 51.8% who reported already having been vaccinated; these proportions decreased with decreasing educational level. Similarly, adults with the highest household incomes were most likely to report being vaccinated or definitely intending to get vaccinated (64.2%), including 42.9% who were already vaccinated; these proportions also decreased with income. Reported COVID-19 vaccination coverage or definite intent to get vaccinated was lower among non-Hispanic Black adults (40.1%, with 25.4% vaccinated) than among non-Hispanic White adults (51.8%, with 35.0% vaccinated). A higher percentage of adults living outside metropolitan areas reported that they probably or definitely would not get vaccinated (40.1%), compared with those within metropolitan areas (22.1%).

Among adults aged 18–39 years, reasons for not intending to get a COVID-19 vaccine varied by vaccine intent (Table 2). Persons who were unsure about getting vaccinated or probably going to get vaccinated, as well as those who were not planning to get vaccinated, had similar levels of concern about experiencing vaccine side effects (56.2% and 56.3%, respectively). Among those who were unsure about getting vaccinated or probably going to get vaccinated, wanting to wait and see if the vaccine was safe (52.9%) and thinking that others needed a vaccine more than they did (39.5%) were the next most frequently cited reasons for not getting vaccinated, whereas lack of trust in COVID-19 vaccines (56.5%) and not believing that a vaccine was necessary (36.4%) were frequently cited reasons among adults aged 18–39 years who were probably or definitely not planning to get vaccinated. Persons who were unsure or probably going to get vaccinated reported a higher level of concern about getting COVID-19 (42.7%) than those who were not planning to get vaccinated (26.1%). Persons who were unsure or probably going to get vaccinated reported that they would be motivated to get vaccinated if they had more information indicating that the vaccines were safe (39.0%), were effective (28.8%), would prevent them from spreading COVID-19 to family and friends (27.6%), and would allow them to resume social activities (20.9%) (Figure). Among those who were unsure or probably going to get vaccinated and those who were not planning to get vaccinated, approximately 60%–70% reported that they were unsure about or did not have enough information about vaccine safety or about vaccine effectiveness (Table 2).

**TABLE 2:** COVID-19 vaccination attitudes and perceptions among adults aged 18–39 years, by vaccination and intent status — United States, March–May 2021

Attitudes and perceptions	Weighted % (95% CI)		
	Vaccination and intent status		
	Vaccinated or definitely planning to get vaccinated (N = 1,521)	Unsure or probably will get vaccinated (N = 562)	Probably or definitely will not get vaccinated (N = 643)
**Reason for not intending to get vaccinated**			
Concerned about possible side effects	NA	56.2 (51.3–61.1)	56.3 (50.9–61.5)
Plan to wait and see if it is safe and might get it later	NA	52.9 (47.4–58.3)	31.2 (26.5–36.2)
Think other people need it more than I do right now	NA	39.5 (34.8–44.3)	14.1 (11.0–17.8)
Concerned about having an allergic reaction	NA	23.5 (18.9–28.6)	23.4 (19.6–27.5)
Do not know if it will work	NA	19.0 (15.1–23.4)	29.3 (24.1–35.0)
Do not trust COVID-19 vaccines	NA	18.0 (14.1–22.3)	56.5 (51.7–61.2)
Concerned about the cost	NA	8.9 (5.9–12.9)	2.6 (1.4–4.5)
Do not believe I need a vaccine	NA	7.2 (4.7–10.6)	36.4 (31.8–41.2)
Do not think COVID-19 is that big of a threat	NA	6.7 (4.2–10.0)	27.4 (23.4–31.7)
**Concern about COVID-19**			
Somewhat/Very concerned about getting COVID-19	53.4 (50.2–56.5)	42.7 (37.8–47.7)	26.1 (21.8–30.8)
**Mask-wearing behavior**			
Always or often wore a mask in public during the past week	95.4 (93.4–96.9)	89.5 (86.3–92.2)	66.5 (61.6–71.2)
**Adequacy of COVID-19 vaccine information **			
Unsure/Not enough information about safety of vaccines	22.2 (19.6–25.0)	71.0 (66.0–75.7)	68.5 (63.3–73.4)
Unsure/Not enough information about how well vaccines protect you	24.2 (21.5–27.1)	67.7 (63.0–72.1)	62.5 (57.1–67.5)
Unsure/Not enough information about where to get a vaccine	22.4 (19.6–25.3)	46.4 (41.4–51.4)	30.0 (25.4–34.8)
**Trusted sources for accurate vaccine information **			
CDC	72.9 (69.9–75.8)	44.5 (39.3–49.8)	22.7 (18.6–27.2)
Primary care providers	61.4 (58.1–64.6)	39.0 (33.9–44.3)	23.1 (18.8–27.8)
State health departments	49.6 (46.3–52.8)	28.2 (23.8–33.0)	10.6 (7.7–14.1)
Local health officials	41.9 (38.5–45.3)	24.1 (19.8–29.0)	8.0 (5.7–11.0)
Family and friends	15.7 (13.3–18.4)	21.0 (16.9–25.6)	16.4 (12.6–20.8)
Food and Drug Administration	45.5 (42.5–48.6)	20.1 (16.3–24.4)	9.8 (7.3–12.8)
News sources	19.7 (17.4–22.2)	13.4 (10.1–17.4)	6.2 (3.9–9.2)
Employer	10.3 (8.6–12.4)	4.3 (2.5–6.8)	3.0 (1.8–4.7)
Social media	2.5 (1.6–3.6)	4.2 (2.3–7.0)	3.4 (1.8–5.9)
Religious organizations	2.2 (1.4–3.3)	2.5 (1.3–4.3)	5.2 (3.4–7.6)
**Barriers to vaccination**			
None/It is not difficult	30.4 (24.9–36.3)	33.0 (28.0–38.3)	62.6 (57.3–67.6)
Do not know where to go to get vaccinated	6.8 (4.3–10.1)	9.5 (7.0–12.7)	2.1 (1.0–3.8)
It is difficult to find or make an appointment	16.4 (12.7–20.6)	8.9 (6.2–12.2)	2.1 (1.1–3.6)
Too busy to get vaccinated	1.5 (0.6–3.0)	7.6 (4.9–11.0)	4.9 (2.8–8.1)
Do not have time off work	5.5 (3.3–8.6)	6.7 (4.0–10.4)	2.3 (1.1–4.4)
The lines are too long	2.3 (1.2–4.1)	4.6 (2.9–7.0)	1.5 (0.7–2.9)
It is too far away or I do not have transportation	4.1 (2.2–7.1)	3.1 (1.4–5.8)	1.2 (0.4–2.5)

**Abbreviations:** CI = confidence interval; NA = not applicable.

**FIGURE:**
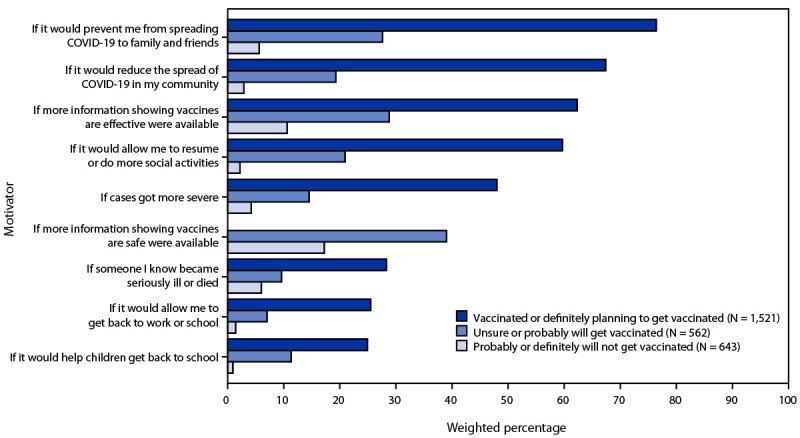
Motivators* for COVID-19 vaccination among adults aged 18–39 years, by intent status — United States, March–May 2021 * Respondents who reported that they had received a COVID-19 vaccine or definitely planned to get vaccinated were asked what made them definitely plan to get vaccinated; all other respondents were asked what would make them more likely to get a COVID-19 vaccine. Weighted percentages represent respondents who chose the motivator in answer to the question, “Which of the following made you definitely plan/would make you more likely to get a COVID-19 vaccine?” The response “more information showing vaccines are safe” was not provided as an option for respondents who reported being vaccinated or who definitely planned to get vaccinated.

Among persons who were unsure about getting vaccinated or probably going to get vaccinated and those who were probably or definitely not going to get vaccinated, the most frequently reported trusted information sources were CDC (44.5% and 22.7%, respectively) and primary health care providers (39.0% and 23.1%, respectively), whereas employers (4.3% and 3.0%, respectively), social media (4.2% and 3.4%, respectively), and religious organizations (2.5% and 5.2%, respectively) were the least frequently reported sources (Table 2). Percentages of persons who reported barriers to vaccine access were generally low (<10%); difficulty making appointments (8.9%) and being too busy to get vaccinated (7.6%) were reported by respondents who were unsure or probably going to get vaccinated. Although 46.4% of these persons reported a lack of adequate information about where to get vaccinated, a much smaller percentage (9.5%) cited this as a barrier to vaccination.

## Discussion

During March–May 2021, nearly one fourth of adults aged 18–39 years were unsure about whether to receive a COVID-19 vaccine or were probably going to get vaccinated, and nearly one fourth reported that they would probably not or definitely not get vaccinated. Among adults aged 18–39 years, those who were younger, were non-Hispanic Black, had lower incomes and educational attainment, had no health insurance, and lived outside of metropolitan areas had the lowest reported vaccination rates and intent to get vaccinated. 

The findings in this report indicate that trust in COVID-19 vaccines, particularly in their safety and effectiveness, was an important factor in the decision to get vaccinated among adults aged 18–39 years, especially for those who were unsure about or probably planning on getting vaccinated. Compared with those who were probably or definitely not planning to get vaccinated, this group was more concerned about getting COVID-19, indicating that information about vaccine safety and effectiveness might have influenced their decision to get vaccinated. This information might be a motivating factor if it were to come from trusted sources, such as health authorities, primary health care providers, and family and friends. In contrast, vaccine messages from employers, religious leaders, or social media might not be as effective. Adults aged 18–39 years who were unsure about getting vaccinated or probably going to get vaccinated reported that a desire to protect others and resume social activities were motivators to get vaccinated, suggesting that messages emphasizing that vaccination would allow them to resume social activities and encouraging vaccination for the greater good might be effective. Ensuring that vaccines are easily accessible, convenient, and available in places where young adults live and work could also improve vaccine acceptance and coverage (*9*).

The findings in this report are subject to at least eight limitations. First, although panel recruitment methodology and data weighting were designed to produce nationally representative results, respondents might not be fully representative of the general U.S. adult population. Second, although data were weighted to account for differential nonresponse, low overall response rates might also affect sample representativeness. Third, because of small sample sizes for the age group 18–39 years within individual surveys, data were combined across multiple survey waves for this analysis, which might have minimized recent changes in vaccination coverage and intent status. Fourth, vaccination intent categories were combined in this analysis, which might have minimized distinctions between categories. However, a preliminary analysis of data from a CDC survey found that attitudes, perceptions, and behaviors were similar for each of the categories within a group. Fifth, state-specific vaccine eligibility varied during the data collection period, and some adults might not have been eligible during previous surveys, which might have affected vaccination coverage responses to questions related to attitudes, behaviors, and perceptions. Sixth, attitudes, behaviors, and perceptions might change quickly, and these results might not reflect current COVID-19 vaccine barriers and motivators. Seventh, results were designed to be national estimates, cannot be generalized at state or local levels, and did not include an examination of geographic differences. Finally, results might not be comparable to results from other national polls or surveys because of potential differences in survey methods, sample design, and framing of questions related to vaccination intent.

Achieving high vaccination coverage among adults aged 18–39 years is critical to protect this population from COVID-19 and to reduce community incidence. Increasing confidence in vaccine safety and effectiveness and emphasizing that vaccines are important for preventing the spread of COVID-19 to family and friends and resuming social activities might help increase coverage in this younger adult population, particularly among those who are unsure about whether to get vaccinated (*5*).

**Summary**

**What is already known about this topic?**

Since April 19, 2021, all persons aged ≥16 years have been eligible for COVID-19 vaccination. Vaccination coverage and intent among adults are lowest among those aged 18–39 years.

**What is added by this report?**

Overall, 34% of adults aged 18–39 years reported having received a COVID-19 vaccine. Adults aged 18–24 years, as well as non-Hispanic Black adults and those with less education, no insurance, and lower household incomes, had the lowest reported vaccination coverage and intent to get vaccinated. Concerns about vaccine safety and effectiveness were commonly cited barriers to vaccination.

**What are the implications for public health practice?**

Addressing concerns about COVID-19 vaccine safety and efficacy and emphasizing the role of vaccination in protecting family and friends and resuming social activities might help increase coverage.

## Footnotes

* Both the Ipsos and NORC panel surveys use an address-based sampling methodology that covers nearly all households in the United States, regardless of their telephone or Internet status. Surveys were conducted in English and Spanish, and non-Hispanic Black persons and non-Hispanic “other race” panel members were oversampled to ensure adequate sample size for subgroup analyses by race/ethnicity.

† Panel completion = proportion of all respondents interviewed among all eligible persons ever contacted. https://www.aapor.org/AAPOR_Main/media/publications/Standard-Definitions20169theditionfinal.pdf

§ A preliminary analysis of data from an in-depth CDC survey that was conducted during this time period and examined behavioral factors associated with vaccination intent status found that attitudes, perceptions, and behaviors were similar for each of the categories within a group. Because all respondents might not have been eligible to get a vaccine during previous surveys, persons who had been vaccinated and those who definitely intended to get vaccinated were combined for analysis.

¶ Metropolitan statistical area (MSA) status was determined by U.S. Census block group using the panelist’s address. For a small number of panelists for whom an address was not available, zip codes were used to determine MSA status. https://www.census.gov/programs-surveys/metro-micro.html

** 45 C.F.R. part 46.102(l)(2), 21 C.F.R. part 56; 42 U.S.C. Sect. 241(d); 5 U.S.C. Sect. 552a; 44 U.S.C. Sect. 3501 et seq.
